# The Effect of Whole Blood Lead (Pb-B) Levels on Changes in Peripheral Blood Morphology and Selected Biochemical Parameters, and the Severity of Depression in Peri-Menopausal Women at Risk of Metabolic Syndrome or with Metabolic Syndrome

**DOI:** 10.3390/ijerph17145033

**Published:** 2020-07-13

**Authors:** Magdalena Sylwia Kamińska, Anna Maria Cybulska, Mariusz Panczyk, Irena Baranowska-Bosiacka, Dariusz Chlubek, Elżbieta Grochans, Marzanna Stanisławska, Anna Jurczak

**Affiliations:** 1 Subdepartment of Long-Term Care, Department of Social Medicine, Faculty of Health Sciences, Pomeranian Medical University in Szczecin, 48 Żołnierska St., 71-210 Szczecin, Poland; magdalena.kaminska@pum.edu.pl; 2 Department of Nursing, Faculty of Health Sciences, Pomeranian Medical University in Szczecin, 48 Żołnierska St., 71-210 Szczecin, Poland; grochans@pum.edu.pl (E.G.); stamarz@pum.edu.pl (M.S.); 3 Department of Education and Research in Health Sciences, Faculty of Health Sciences, Medical University of Warsaw, 81 Żwirki i Wigury St., 02-091 Warszawa, Poland; mariusz.panczyk@wum.edu.pl; 4 Department of Biochemistry and Medical Chemistry, Pomeranian Medical University in Szczecin, 72 Powstańców Wielkopolskich St., 70-111, Szczecin, Poland; ika@pum.edu.pl (I.B.-B.); dchlubek@pum.edu.pl (D.C.); 5 Department of Specialized Nursing, Faculty of Health Sciences, Pomeranian Medical University in Szczecin, 70-210 Szczecin, Poland; anna.jurczak@pum.edu.pl

**Keywords:** Pb-B, metabolic syndrome, menopause, depression

## Abstract

The aim of our study was to assess the impact of whole blood lead (Pb-B) levels on changes in peripheral blood morphology and selected biochemical parameters, and the severity of depression in peri-menopausal women at risk of metabolic syndrome (pre-MetS) or with metabolic syndrome (MetS). The study involved 233 women from the general population of the West Pomeranian Province (Poland) aged 44–65 years. The intensity of menopausal symptoms and the severity of depression was examined using the Blatt–Kupperman Index (KI) and the Beck Depression Inventory (BDI). C-reactive protein (CRP), insulin, glucose, glycated hemoglobin (HbA1C), high-density lipoprotein (HDL) and low-density lipoprotein (LDL) cholesterol, triglyceride levels (TG), cortisol, morphology of blood cells and homeostasis model assessment for insulin resistance (HOMA-IR) and Pb-B was measured. Women with MetS had higher levels of glucose, HbA1C, HDL, LDL, TG, cortisol, insulin and higher HOMA-IR. No significant differences in Pb-B were observed between pre-MetS and the control group, and between pre-MetS and the MetS group. A significant correlation was noticed between Pb-B vs. the percentage of monocytes in blood, and blood cortisol levels in women with MetS; Pb-B vs. lymphocyte count and HbA1C in the pre-MetS group, as well as in the BDI scores between the MetS and pre-MetS group. We cannot clearly state that exposure to Pb is an environmental factor that can be considered as a risk factor for MetS in this studied group.

## 1. Introduction

Metabolic syndrome (MetS) involves the coexistence of metabolic risk factors that are linked to one another. They include visceral obesity, atherogenic dyslipidemia in the form of hypertriglyceridemia and decreased HDL cholesterol levels, carbohydrate metabolism disorders (impaired fasting glycemia), carbohydrate intolerance, type 2 diabetes, and hypertension [1]. At present, the criteria proposed by the International Diabetes Federation (IDF), and the modified criteria of the National Cholesterol Education Program-Adult Treatment Panel III (NCEP-ATPIII) are equally binding and present in the specialist literature [1,2]. Metabolic risk factors, included in the above sets of MetS criteria, contribute to the development of atherosclerotic cardiovascular disease [3], type 2 diabetes (if it is not a component of MetS) [4], and neurological disorders [5], and they raise both overall and cardiovascular mortality rate [6,7]. MetS is currently regarded as a pandemic [8]. The results of epidemiological research indicate that MetS is widely spread both across the US [9,10,11], and Europe [12,13,14], including Poland [15,16]. The incidence of MetS increases with age [10,11,13,17], but in some countries, this increase is substantially higher for women than for men [13,15,16]. It should be emphasized, though, that the incidence of MetS differs markedly between populations for various reasons, such as using different MetS definitions and criteria for its diagnosis, or different measurement methods.

Scientific evidence suggests that some mechanisms associated with mitochondrial dysfunction and insulin resistance [18], inflammatory factors [19], and the functioning of the microbiome can contribute to MetS and related diseases [20]. The etiopathogenesis of MetS includes three groups of etiological factors: obesity and disorders of adipose tissue metabolism; insulin resistance and compensatory hyperinsulinemia; and a set of independent risk factors, such as genetic predisposition, the process of aging, hormonal, depressive, and anxiety disorders, chronic stress, sleep deficiency, and environmental factors (a high-calorie and atherogenic diet, low physical activity, etc.) [1]. The processes of aging differs depending on sex, which is related to the functioning of the immune system and the occurrence of metabolic disorders. In many cases, sex-specific differences in the immune responses of young adults are also observed in older men and women. This is probably due to pre-existing differences in immunity between men and women, as well as differences between the course of menopause and andropause. Changes in the hormone profile that occur with age are associated with impaired carbohydrate tolerance, which results from a decrease in insulin secretion and an increase in tissue insulin resistance. Furthermore, the cardiovascular risk associated with MetS has been shown to be highly dependent on gender and sex hormone status. In addition, men and women have an age-related cardiometabolic risk that is gender-related due to changes in sex hormone levels. That is why the concept of MetS is currently considered in the context of a gender-dependent diagnosis. It is reasonable to conduct new research and clinical trials with regard to the role of gender differences in the development of metabolic regulation disorders and cardiometabolic risk [21,22].

In recent years, increasing attention has been paid to the influence of the environment on the occurrence of metabolic disorders. There are publications available that describe the relationship between MetS symptoms and cytotoxicity of heavy metals, especially lead (Pb). The toxicity of Pb is relatively well known. Its hematotoxic effect is associated with anemia [23]. The effects of Pb hepatotoxicity are lipid peroxidation and a substantial increase in the parameters of hepatic function, accompanied by the suppression of antioxidant enzyme activity [24,25]. The nephrotoxicity of Pb manifests as impaired kidney functioning and Fanconi’s syndrome [26]. Exposure to Pb entails the risk of immunotoxicity resulting from partial immunosuppression and deregulation of the immune system, and oversensitivity in the form of allergy, anaphylaxis, or autoimmune and autodegenerative diseases [27].

It has been demonstrated that there is a relationship between Pb accumulation in adults and the incidence of hypertension and cardiovascular disease [28,29,30]. Some authors have reported that even a low level of environmental Pb exposure is an important predictor of the risk of cardiovascular death in the US [31]. What is more, high blood Pb levels have been shown to be significantly associated with the risk of MetS [32,33]. A relationship between even very low whole blood lead (Pb-B) levels and nervous system disorders has been proven [34,35,36,37,38]. In addition, Pb can contribute to the development of mental disorders (especially depression) through mechanisms causing irregularities in the serotonergic system (5-HT), hypothalamic–pituitary–adrenal (HPA) axis dysregulation, and an increase in corticotropin-releasing hormone (CRH) and cortisol levels [39,40,41,42]. 

In the course of the peri-menopausal period, estrogen production gradually decreases and eventually stops. This period involves a number of vasomotor symptoms, as well as fatigue and apathy, impaired concentration, attention, and memory, sleep disorders, lower libido, mood disorders, and depression [43]. Scientific reports indicate that depression is common in peri-menopausal women, which has serious health, social and economic consequences [44,45,46]. Furthermore, MetS is more commonly observed among people with a history of depressive symptoms, especially those with current major depression, although this relationship is not associated with age and sex [47]. To our knowledge, few studies have been conducted to assess the association of toxic heavy metals (especially Pb) with cardiovascular risk factors, including visceral obesity, hypertension, dyslipidemia and carbohydrate metabolism disorders in peri-menopausal women, especially those showing metabolic syndrome symptoms.

Therefore, the aim of our study was to assess the impact of whole blood lead (Pb-B) levels on changes in peripheral blood morphology and selected biochemical parameters, as well as the severity of depression in peri-menopausal women at risk of metabolic syndrome (pre-MetS) or with metabolic syndrome (MetS).

## 2. Materials and Methods

The research was carried out in the West Pomerania Province of Poland in a group of 233 women from the general population.

The inclusion criteria were as follows: female sex, age between 44–65 years, deliberate written consent to take part in the study. The exclusion criteria: inflammatory, psychiatric or cancerous diseases.

The study was carried out in accordance with the Helsinki declaration after obtaining the approval of the Bioethical Commission of the Pomeranian Medical University in Szczecin (covered for blind review; permission numbers KB-0012/181/13). This was a cross-sectional non-random study using convenience sampling. Recruitment was performed based on information posters in public places and advertisements in local papers.

### 2.1. Study Design

The research was carried out in several stages. The methods applied in this study were: diagnostic survey; the measurement of waist, hips, and blood pressure; and laboratory methods. In the first step, we used the author’s questionnaire, covering a number of questions regarding basic sociodemographic data (age, place of residence, professional activity, education, marital status), stimulants (alcohol, caffeine, tobacco), and health (menstruation, inflammatory status, psychiatric and cancerous diseases, menopausal status). Menopausal status was categorized as peri-menopause (climacteric transition with irregular menses) and menopause (last menstrual period at least 12 months prior to the survey).

We used two standardized questionnaires: The Blatt–Kupperman Index (KI) and the Beck Depression Inventory (BDI).

The KI is an 11-item questionnaire used to assess climacteric symptoms. It was developed by H.S. Kupperman and M.H. Blatt in 1952. The questionnaire includes somatic symptoms such as sweating/hot flushes, palpitation, vertigo, headache, paresthesia, formication, arthralgia, and myalgia, and psychological symptoms such as fatigue, nervousness, and melancholia. The severity of these complaints is rated on a 0–3 scale. The total score is the sum of all items. Scores are interpreted as follows: 0–16 = no symptoms, 17–25 = mild symptoms, 26–30 = moderate symptoms, and ≥31 = severe symptoms [48].

The BDI is a self-reporting questionnaire for measuring the severity of depression in normal and psychiatric populations. Created by Aaron T. Beck in 1961, the BDI consists of 21 items rated on a 4-point scale (from 0 = no symptoms to 3 = severe symptoms). The total score is the sum of the highest ratings for all 21 items. The minimum score is 0 and maximum score is 63—the higher the score, the more severe the symptoms. In non-clinical populations, scores above 20 indicate depression. In those diagnosed with depression, scores of 0–11 = minimal depression, 12–19 = mild depression, 20–25 = moderate depression and ≥26 = severe depression [49].

The next stage involved anthropometric measurements, such as waist and hip circumference. Waist and hip circumference were measured to the nearest 0.01 m using a flexible measuring tape (Seca). Waist circumference (WC) was measured as the horizontal distance around the abdomen at the umbilicus level. Hip circumference (HC) was measured as the distance passing horizontally through the two superior iliac bones. Additionally, the waist/hip ratio (WHR) was determined as an indicator of abdominal obesity. Values above 0.9 were regarded as abnormal, but this indicator was not used to determine abdominal obesity. Abdominal (central) obesity was diagnosed if WC ≥ 80 cm (for European women) [50].

The Korotkoof sound technique was applied to measure blood pressure (BP). We ensured the correct position of the patient, a period of calm rest, the use of a cuff of the right size, and minimization of external factors affecting blood pressure, such as smoking and taking caffeine-containing products before measuring blood pressure [51]. We followed the recommendations of the American Heart Association [52].

Next, venous blood was collected from each of the volunteers after overnight fasting between 7.00 a.m. and 9.30 a.m. in the morning after a 10 min rest in a sitting position from the antecubital vein using Vacutainer tubes free of Pb (Sarstedt, Germany). Two tubes were collected: one for lead level in whole blood (Pb-B) measurement (3 mL) and the other for the biochemical analysis of serum and for blood morphology (7 mL).

The blood was collected in accordance with the relevant rules and procedures concerning collecting, storing, and transporting biological material. C-reactive protein (CRP), insulin, glucose, glycated hemoglobin (HbA1C), total, high-density lipoprotein (HDL) and low-density lipoprotein (LDL) cholesterol, triglyceride levels (TG), cortisol, morphology of blood cells, lead level in whole blood (Pb-B) and homeostasis model assessment for insulin resistance (HOMA-IR) were determined. The women were divided into three groups:

- group I (MetS): women with MetS;
- group II (pre-MetS): women at risk of MetS;
- group III (control): women without MetS and the risk of MetS.

### 2.2. Definitions

#### 2.2.1. Definition of Metabolic Syndrome (MetS)

Women were considered to have MetS if they had any three of the following five risk factors according to criteria by the International Diabetes Federation (IDF) and the American Heart Association/National Heart, Lung, and Blood Institute (AHA/NHLBI), 2009:

- waist circumference ≥ 80 cm in females, raised TG level > 150 mg/dL (1.7 mmol/L), or specific treatment for this lipid abnormality;
- reduced HDL cholesterol: < 50 mg/dL (1.3 mmol/L) in females, or specific treatment for this lipid abnormality;
- raised blood pressure (BP): systolic BP ≥ 130 or diastolic BP ≥ 85 mmHg, or treatment of previously diagnosed hypertension;
- raised fasting plasma glucose (FPG) ≥ 100 mg/dL (5.6 mmol/L), or previously diagnosed type 2 diabetes. If above 5.6 mmol/L or 100 mg/dL, oral glucose tolerance test (OGTT) is strongly recommended but is not necessary to define the presence of the syndrome [1,2].

#### 2.2.2. Definition of Pre-Metabolic Syndrome (Pre-MetS)

Pre-metabolic syndrome (pre-MetS) was defined as having at least two components of MetS, but not meeting the above criteria for the diagnosis of MetS [53,54].

### 2.3. Determination of Biochemical Parameters and Lead Levels

The determination of biochemical parameters was performed at a certified laboratory in the Pomeranian Medical University in Szczecin using standardized commercial methods.

Whole blood lead (Pb-B) levels were determined by means of inductively coupled plasma optical emission spectrometry (ICP-OES, ICAP 7400 Duo, Thermo Scientific), using a concentric nebulizer and a cyclonic spray chamber. Analysis was performed in axial mode.

The samples were mineralized using the CEM MARS 5 (CEM Corporation, Matthews, US) microwave digestion system. One milliliter of each blood sample was taken into a clean polypropylene tube and 1 mL of 65% HNO_3_ was added to each vial. Each sample was allowed 30 minutes pre-reaction time in a clean hood. Next, 1 mL of non-stabilized 30% H_2_O_2_ solution [55,56] was added to each vial. The samples were then placed in special Teflon vessels and heated in the microwave digestion system for 35 minutes at 180 °C (15 min ramp up to 180 °C, then maintained at 180 °C for 20 min). After digestion, all samples were removed from the microwave and allowed to cool down to room temperature. In a clean hood, the samples were transferred to acid-washed 15 mL polypropylene tubes and stored in a monitored refrigerator at a temperature of 4 °C until analysis.

A 5-fold dilution was performed prior to ICP-OES measurement. The samples were spiked with an internal standard to provide a final concentration of 0.5 mg/L Yttrium, 1 mL of 1% Triton (Triton X-100, Sigma), and diluted to the final volume of 6 mL with 0.075% nitric acid. Blank samples were prepared by adding concentrated nitric acid to tubes without samples, and then diluting as described above. Multielement calibration standards (ICP multi-element standard solution IV, Merck) were prepared with different concentrations of inorganic elements in the same manner as blanks and samples. All solutions were prepared using deionized water (Direct Q UV, Millipore, approximately 18.0 MΩ). The wavelength for Pb was 220.353 nm.

The level of 5 μg/dL Pb-B is regarded as a ‘threshold level’ for pregnant women and children, and the level of 10 μg/dL Pb-B is considered as ‘safe’ for adults [57,58,59,60].

### 2.4. Statistical Analysis

Statistical analysis was performed in the TIBCO^®^ Software Statistica™ version 13.3 (Palo Alto, California, USA). Descriptive statistics—arithmetic mean (M), minimum (Min), maximum (Max), standard deviation (SD), quartile (Q)—were used to describe quantitative variables.

Qualitative variables were presented using number (n) and percent (%). The chi-square (chi^2^) test was applied to compare qualitative variables, and Fisher’s test was employed when low expected values appeared in the tables.

Quantitative variables in three or more groups were compared using analysis of variance (ANOVA) in cases of normal distribution, and the Kruskal–Wallis test was applied if normal distribution was not observed. Post–hoc analysis was performed in cases of statistically significant differences. We used Fisher’s Least Significant Difference (LSD) test if normal distribution was confirmed, and Dunn’s test if a variable was not normally distributed. The normality of the variable distribution was assessed using Shapiro–Wilk’s test. The Pearson’s correlation rank coefficient was employed to determine the strength of the correlations between the parameters.

The effects with a probability value (p) lower than the significance level of 0.05 (*p* < 0.05) were assumed significant.

## 3. Results

A total of 233 menopausal women were studied. Table 1 shows the baseline characteristics of these women with MetS (n = 47, 20.2%), pre-MetS (n = 64, 27.4%) and the control group (n = 122, 52.3%). The mean age of our subjects was 53.23 ± 5.41 years.

Analysis of the selected biochemical parameters of the studied women with regard to their MetS status confirmed that women with MetS had higher mean levels of glucose, TG, HDL, LDL, HbA1C, cortisol, insulin, and higher HOMA-IR, which is in line with the definition of MetS (Table 2).

Post hoc analysis demonstrated that there were no statistically significant differences in mean Pb-B levels between the pre-MetS and the control group (Tukey’s HSD test: *p* = 0.159), and between the pre-MetS vs. MetS group (Tukey’s HSD test: *p* = 0.066). However, there was a statistically significant difference in the mean Pb-B levels between the control group and the group with MetS; the level of this element was higher in the control group (Tukey’s HSD test: *p* = 0.001) (Table 2). All groups had Pb-B levels below the ‘threshold level’ for adults (Table 2).

Analysis of the selected biochemical and anthropometric parameters of the studied women with regard to their MetS status revealed a statistically significant relationship between MetS and Pb-B levels. The mean Pb-B level in women with MetS was significantly negatively correlated to the percentage of monocytes in their blood (r = −0.33; *p* = 0.033) and positively correlated with blood cortisol levels (r = 0.36; *p* = 0.019). What is more, there was a statistically significant positive correlation between the level of Pb-B in women with pre-MetS and the lymphocyte count (r = 0.26; *p* = 0.049) and HbA1C (r = 0.36; *p* = 0.005). In the control group, a positive significant correlation between Pb-B levels and waist circumference was observed (r = 0.27; *p* = 0.004) (Table 3).

The vast majority of the studied women did not menstruate, irrespective of which group they belonged to (n = 151, 64.8%; Table 4). Analysis did not demonstrate a statistically significant difference in Pb-B levels between menstruating and non-menstruating women (ANOVA: F = 0.717; *p* = 0.398). The differences in mean Pb-B levels between the three groups of women (MetS vs. pre-MetS vs. control) were similar in menstruating and non-menstruating women (two-way ANOVA: F = 0.632; *p* = 0.532; Table 5).

The vast majority of the studied women declared using stimulants, irrespective of which group they belonged to. No statistically significant relationship was observed between Pb-B levels and using stimulants (ANOVA: F = 0.009; *p* = 0.926). The differences in Pb-B levels between the three groups of women (MetS vs. pre-MetS vs. control) were similar in users and non-users of stimulants (two-way ANOVA: F = 0.347; *p* = 0.707; Table 6).

One aspect analyzed in our study was the significance of differences between MetS status and the severity of climacteric symptoms as assessed by the KI. No statistically significant differences was found (*p* > 0.05; Table 7).

Another aspect analyzed in our study was the significance of differences in MetS status and the severity of depressive symptoms as assessed by the BDI. A statistically significant difference was noted in the BDI results (ANOVA: F = 3.686; *p* = 0.031; η^2^ = 0.031; Table 7). Post hoc analysis revealed that there were no statistically significant differences in the mean severity of depressive symptoms between the control group and MetS group (Tukey’s HSD test: *p* = 0.208), and between the control group and pre-MetS group (Tukey’s HSD test: *p* = 0.293). There was a statistically significant difference in the mean BDI scores between the group with MetS and the pre-MetS group, with more severe depressive symptoms observed in the pre-MetS group (M: 6.04 vs. 9.70; Tukey’s HSD test: *p* = 0.018). There was no statistically significant correlation between Pb-B levels and depressive symptoms with regard to MetS status (Table 8).

## 4. Discussion

In current literature, there have been few studies showing the relationship between MetS in the general population (especially in peri-menopausal women) and heavy metals concentration the human body, especially lead. This heavy metal triggers oxidative stress through binding to sulfhydryl groups of proteins, causing inactivation of numerous enzymatic reactions and amino acids, and depletion of antioxidants [61,62]. These mechanisms can lead to the development of cardiovascular disease, diabetes, atherosclerosis, neurological disorders, and chronic inflammation [61]. The Chinese study demonstrated that the ratio of apolipoprotein B to apolipoprotein A1 is a good predictive indicator of MetS and pre-MetS in young women with polycystic ovary syndrome [53]. Some studies suggest that oxidative stress contributes to insulin resistance, playing an important role in the pathogenesis of MetS [63,64,65]. There are several publications concerning the influence of particular toxic heavy metals on the cardiovascular and metabolic systems. Moon’s study showed a positive correlation between Pb-B and elevated TG levels and increased waist circumference [62]. In another study, MetS components such as hypertension, dyslipidemia, and dysglycemia were not correlated with Pb-B levels [61]. However, further research proved the combined toxicity of heavy metals (lead, mercury, and cadmium) even if they are below toxic levels. The authors of this study showed that tbe accumulation of each heavy metal that impairs the functioning of the endocrine system may have an additive or synergic effect on the development of particular metabolic disorders, even if this heavy metal alone does not show such an effect [61].

In our study, analysis of the selected biochemical and anthropometric parameters of the studied women with regard to their MetS status demonstrated a statistically significant relationship between MetS and Pb-B levels. We showed that women with MetS had higher mean levels of glucose, TG, HDL, LDL, HbA1C, cortisol, insulin, and higher HOMA-IR, which is in line with the definition of MetS. Czech studies have shown that the transition to menopause increases the cardiovascular risk associated with MetS. The higher incidence of MetS is not only due to a marked increase in visceral obesity assessed on the basis of waist circumference and triglyceride levels, but also due to an increase in insulin resistance index (HOMA-IR), which reaches the highest values during menopause in the population of women with the highest risk of MetS. Researchers suggest that women who enter the menopausal period with isolated MetS components are at high risk of MetS manifestation, and it is in relation to them that preventive measures should be taken in this regard [66].

In our study, all groups had Pb-B levels below the ‘threshold level’ for adults. There were no statistically significant differences in the mean Pb-B levels between the pre-MetS and the control groups or between the pre-MetS and the MetS groups. However, there was a statistically significant difference in the mean Pb-B levels between the control group and the group with MetS; the level of this element was higher in the control group. It is difficult to explain this fact explicitly. The vast majority of examined women were employed and lived in large urban agglomerations. The influence of additional sociodemographic risk factors on the increase in Pb-B levels cannot be excluded. For this reason, we cannot clearly state that Pb-B may be a predictor of the risk for pre-MetS. There are few epidemiological data available on Pb-B levels in Poland. Some reviews showed highly varied levels of Pb pollution in different regions of Poland—in large industrial cities, average Pb-B concentrations were usually twice as high as in rural areas of the studied region [67]. In another study the values of Pb-B decreased with a distance from the source of Pb emission [68]. It is worth noting that in general, biomonitoring data on the Pb-B levels in Polish peri-menopausal women were not found in the available literature.

However, our study revealed a significant negative correlation between the level of Pb-B in women with MetS and the percentage of monocytes in their blood, as well as a positive correlation with their blood cortisol levels. A significant positive correlation was also found between Pb-B levels in women at risk of MetS and their lymphocyte count. This may suggest a negative effect of Pb on the immune system, even if the levels of this element were previously considered acceptable (below 10 µg/dL). In addition, we showed a positive correlation between glycated hemoglobin and Pb-B levels in the group of women at risk of MetS in our study. This is especially important because MetS raises the risk of cardiovascular disease, cardiovascular mortality, and the risk of developing type 2 diabetes [69,70,71,72]. MetS is associated with subclinical atherosclerosis in women without symptoms of diabetes as early as in the first decade after menopause [73]. The triglyceride–glucose (TyG) index, on the other hand, is related to carotid atherosclerosis and arterial stiffness, mainly in slim post-menopausal women, whereas MetS is a better predictor of subclinical atherosclerosis in women who are overweight or obese [74].

In our study, the vast majority of women did not menstruate, irrespective of their MetS status. No statistically significant relationship was observed between menstruation status and the level of Pb-B. However, one study showed that higher prenatal and early childhood exposure to Pb may be associated with delayed pubertal development of girls [75]. Pb-B levels above 5 μg/dL were related to a delay in menarche in 13-year-old South African girls [76]. At the same time, another two studies provided no evidence for similar negative associations in US girls at the age of nine [77], or in Polish girls between 7–16 years of age [78]. It has been suggested that early exposure to Pb can result in the decreased levels of pubertal hormones, and consequently in delayed pubertal development [79,80]. No data have been found on the relationship between the last menstruation and blood Pb levels in peri-menopausal women.

As available studies indicate, the incidence of MetS among women depends on their menopausal status (pre- and post-menopausal periods) and is associated with involution hormonal changes and related changes in body composition [81,82]. With age, a decrease in lean body mass, a decrease in skeletal muscle mass, an increase in the percentage of adipose tissue, as well as a decrease in basal metabolism and total energy expenditure are observed [83]. Three independent meta-analyses demonstrated that the incidence of MetS in post-menopausal women is relatively high and considerably higher than in pre-menopausal women [84,85,86]. In addition, a higher probability of diagnosing MetS in post-menopausal women than in their pre-menopausal counterparts has been demonstrated, based on the assessment of MetS components, regardless of the type of MetS diagnostic criteria [85]. It is suspected that MetS may also be associated with post-menopausal hyperandrogenism in women. However, the study by Meun et al. did not confirm the relationship between high androgen levels as a residue of previous polycystic ovary syndrome in serum and an increased risk of cardiovascular disease [87].

The vast majority of the surveyed women declared that they used stimulants, regardless of the groups they belonged to. However, analysis did not show a statistically significant difference in mean Pb-B levels between stimulant users and non-users. The differences in mean Pb-B levels between the three groups of women (MetS vs. at risk of MetS vs. control) were similar in stimulant users and non-users. A statistically significant relationship was observed between using stimulants and MetS status. Meanwhile, the authors of a Norwegian population study found that low physical activity and heavy smoking increase the incidence of MetS in both women and men [88]. A large multicenter study of post-menopausal women, based on multidimensional logistic analyses, showed that MetS was associated with age, the elapsed time from menopause, smoking, obesity, and hypertension [89]. Moreover, a meta-analysis by Sun et al. showed that active smoking is associated with the development of MetS [90]. A study by American authors, on the other hand, showed that alcohol consumption by both women and men is associated with the presence of MetS components, such as low serum HDL cholesterol, elevated serum triglycerides, high waist circumference, and hyperinsulinemia [91]. The meta-analysis conducted by Sun et al. shows that an increased risk of MetS may be related to drinking large amounts of alcohol, and opposite, moderate alcohol intake seems to be related to a lower risk of MetS [92].

Our investigation did not reveal any statistically significant differences in the severity of climacteric symptoms as assessed by the KI between the group at risk of MetS and the control group, or between the group at risk of MetS and the group with MetS. According to the study conducted by Cengiz et al. on the basis of the Menopause Rating Scale, sleep problems, depressed mood, irritability, anxiety, physical and mental fatigue, as well as problems with defecation are much more common in women with MetS, but no difference in the above vasomotor symptoms was found between patients with and without MetS [93]. As indicated by other studies, women experiencing hot flashes or night sweats have an increased risk of developing ischemic heart disease in the next 10-15 years [94]. In addition, there is evidence linking vasomotor symptoms to insulin resistance, abnormal lipid levels, obesity, and type 2 diabetes [95,96].

Our study demonstrated statistically significant differences in the BDI results. Post hoc analysis showed that there were no statistically significant differences in the mean severity of depressive symptoms between the control group and the group with MetS, or between the control group and the group at risk of MetS. However, there was a statistically significant difference in the mean BDI scores between the group with MetS and the group at risk of MetS, with higher severity observed in the group at risk of MetS. The systematic review and meta-analysis performed by Iranian authors confirm the association between depression and MetS [97]. The prospective research conducted by Räikkönen et al. to assess the effect of psychosocial factors related to cardiovascular disease and type 2 diabetes on the risk of MetS in pre-menopausal women provided evidence that women who reported severe depressive symptoms and experienced very stressful life events at the beginning of the study had a higher risk of developing MetS during a 15-year observation, on average. Furthermore, there was a relationship between the psychosocial characteristics and health behaviors of the studied women—a low level of physical activity and smoking were correlated with depression, and a higher alcohol intake was associated with anger, anxiety, and stress [98].

In our study, there was no statistically significant correlation between Pb-B levels and depressive symptoms with regard to MetS status. Meanwhile, our previous research has shown that exposure to Pb may be a factor in the development of depressive symptoms in menopausal women. It may also be associated with glucose metabolism disorders and immunosuppression in women during this period of life [99]. What is more, Chinese research has demonstrated the relationship between Pb-B levels and depression in the elderly population. It has been shown that the risk of depression rises with the increase in Pb-B levels, and this relationship is more pronounced in rural areas than in urban areas due to higher Pb-B levels in people living in rural areas [100]. Another study suggests that depression can lead to the development of cardiovascular disease through its association with MetS, and that depressive symptoms can be a consequence and not the cause of MetS, since obesity and dyslipidemia have been shown to be predictors of depressive symptoms. The authors noted that the relationship between MetS and depression seems to be bidirectional [101]. A systematic review and meta-analysis of epidemiological studies also demonstrated a two-way relationship between depression and MetS in adults. It was found that depression and MetS were significantly correlated in cross-sectional studies, while a bidirectional association was observed in prospective cohort studies [102].

In our research, Pb-B levels were related to changes in blood counts (the number of monocytes and lymphocytes) and to disturbed biochemical parameters (glucose metabolism and cortisol level) in peri-menopausal women at risk of MetS and with MetS. However, we found no connection between Pb-B and the severity of depression in the studied women with MetS. Nevertheless, a negative effect of Pb on the severity of depression was observed in the group of women at risk of MetS. This observation is extremely important from a diagnostic point of view, and we believe that women at risk of MetS should be covered by preventive measures in this regard. The results of our research did not provide unequivocal evidence that environmental exposure to Pb, even at low concentrations (below the ‘threshold level’ for adults), can be considered as a risk factor for MetS in peri-menopausal women.

## 5. Limitations

We only took into consideration the general information provided by the participants on the use of stimulants. No separate questions about alcohol, caffeine or tobacco were asked, and no detailed analysis of particular stimulants used by the participants was performed. Nonetheless, our goal was to assess the problem of stimulants in general. We were also concerned that participants might hide the truth when more specific questions were asked, so we decided that general questions about stimulants would provide more reliable results.

The study design does not take liver function parameters into account. This is a major limitation, considering that non-alcoholic fatty liver disease (NAFLD) is an important MetS correlate.

Our study is not randomized, so the results of the study cannot be regarded as representative.

## 6. Conclusions

Considering the above, we cannot clearly state that environmental exposure to Pb even at low concentrations (below the ‘threshold level’ for adults) can be considered as a risk factor for MetS or depression in the studied group of peri-menopausal women. It is recommended that MetS prevention in peri-menopausal women involves a comprehensive health assessment, with particular emphasis on risk factors for cardiovascular disease and type 2 diabetes, as well as psychosocial factors, which should be considered in relation to all aspects of overall body function.

## Figures and Tables

**Table 1:** Baseline data of menopausal women with metabolic syndrome (MetS), pre-metabolic syndrome (pre-MetS) and the control group.

Parameters	MetS (n = 47)		Pre-MetS (n = 64)		Control (n = 122)		All (n = 233)	
	M	SD	M	SD	M	SD	M	SD
Age (years)	54.49	5.65	53.06	5.16	52.84	5.42	53.23	5.41
WC (cm)	96.98	11.35	92.89	9.52	81.24	11.18	87.70	12.73
HC (cm)	106.77	10.41	105.78	7.99	98.01	9.42	101.96	10.10
WHR (cm)	0.91	0.07	0.88	0.07	0.83	0.1	0.86	0.09
SBP (mmHg)	122.62	18.92	120.08	12.61	109.39	11.36	115.12	14.77
DBP (mmHg)	80.74	11.08	81.00	7.83	72.97	7.78	76.83	9.41

MetS: group with metabolic syndrome; Pre-MetS: group with risk of metabolic syndrome; WC: waist circumference; HC: hip circumference; WHR: waist to hip ratio; SBP: systolic blood pressure; DBP: diastolic blood pressure; M: mean; SD: standard deviation.

**Table 2:** Biochemical parameters of the studied women with regard to their metabolic syndrome status.

Parameters	MetS (n = 47)		Pre-MetS (n = 64)		Control (n = 122)		All (n = 233)		F_(2, 228)_	*p*-Value *
	M	SD	M	SD	M	SD	M	SD		
FPG (mg/dL)	100.96	40.37	84.09	9.41	81.79	7.86	86.31	20.89	18.6 8	0.000
TG (mg/dL)	201.31	203.89	133.76	300.24	82.54	26.94	120.73	188.07	7.145	0.001
Total Cholesterol (mg/dL)	235.41	50.12	220.04	46.31	210.89	34.33	218.38	42.27	5.790	0.004
HDL (mg/dL)	56.10	15.74	68.89	14.39	74.09	17.94	69.01	17.90	19.742	0.000
LDL (mg/dL)	145.61	43.56	127.12	27.83	120.25	31.49	127.21	34.58	9.550	0.000
HbA1C [%]	5.65	1.09	5.30	0.31	5.22	0.25	5.33	0.57	11.065	0.000
Cortisol [µg/dL]	15.30	5.99	13.75	4.88	14.53	9.70	14.47	7.93	0.375	0.688
Insulin [µlu/ml]	13.95	6.89	11.14	6.38	7.75	5.21	9.94	6.39	20.817	0.000
HOMA-IR	3.63	2.99	2.37	1.52	1.59	1.21	2.20	1.93	22.247	0.000
Pb-B [µg/dL]	5.64	2.03	6.99	2.71	8.01	4.38	7.25	3.70	8.627	0.000

MetS: group with metabolic syndrome; Pre-MetS: group with risk of metabolic syndrome; FPG: fasting plasma glucose; TG: triglyceride; HDL: high-density lipoprotein; LDL: low-density lipoprotein; HbA1C: glycated hemoglobin; HOMA-IR: homeostatic model assessment of insulin resistance; M: mean; SD: standard deviation; F: statistical value; *p*-value: significance level; * one-way ANOVA.

**Table 3:** Correlation between anthropometric and biochemical parameters and Pb-B level (µg/dL) with regard to metabolic syndrome status.

Parameters	MetS (n = 47)		Pre−MetS (n = 64)		Control (n = 122)	
	r_p_	*p*−Value	r_p_	*p*−Value	r_p_	*p*−Value
Age (years)	0.12	>0.05	0.12	>0.05	0.08	>0.05
WC (cm)	−0.02	>0.05	0.00	>0.05	0.27	0.004
HC (cm)	0.09	>0.05	0.01	>0.05	0.01	>0.05
SBP (mmHg)	−0.12	>0.05	0.03	>0.05	−0.03	>0.05
DBP (mmHg)	0.06	>0.05	−0.04	>0.05	−0.01	>0.05
Leucocytes (thousand/µL)	0.17	>0.05	0.21	>0.05	−0.04	>0.05
Erythrocytes (million/µL)	0.19	>0.05	−0.21	>0.05	0.16	>0.05
Hemoglobin (g/dL)	0.18	>0.05	−0.01	>0.05	0.17	>0.05
Hematocrit (%)	0.18	>0.05	−0.06	>0.05	0.02	>0.05
MCV (femtolitre)	−0.00	>0.05	0.23	>0.05	0.03	>0.05
MCH (pg)	0.02	>0.05	0.24	>0.05	0.02	>0.05
MCHC (g/dL)	0.07	>0.05	0.02	>0.05	−0.00	>0.05
Platelets (thousand/µL)	−0.01	>0.05	0.09	>0.05	−0.03	>0.05
Neutrophils (%)	0.25	>0.05	0.08	>0.05	−0.04	>0.05
Lymphocytes (%)	−0.30	>0.05	0.04	>0.05	0.09	>0.05
Monocytes (%)	−0.33	0.03	−0.19	>0.05	−0.07	>0.05
Eosinophils (%)	0.04	>0.05	−0.12	>0.05	−0.11	>0.05
Basophils (%)	−0.09	>0.05	−0.00	>0.05	0.06	>0.05
Neutrophils (thousand/µL)	0.27	>0.05	0.18	>0.05	−0.04	>0.05
Lymphocytes (thousand/µL)	−0.04	>0.05	0.26	0.05	0.06	>0.05
Monocytes (thousand/µL)	−0.12	>0.05	0.02	>0.05	−0.07	>0.05
Eosinophils (thousand/µL)	0.08	>0.05	−0.01	>0.05	−0.14	>0.05
Basophils (thousand/µL)	0.01	>0.05	0.05	>0.05	0.03	>0.05
FPG (mg/dL)	0.07	>0.05	0.02	>0.05	−0.12	>0.05
TG (mg/dL)	0.03	>0.05	0.20	>0.05	0.03	>0.05
Total Cholesterol (mg/dL)	0.05	>0.05	0.10	>0.05	−0.00	>0.05
HDL (mg/dL)	−0.09	>0.05	0.07	>0.05	0.04	>0.05
LDL (mg/dL)	0.14	>0.05	0.02	>0.05	−0.03	>0.05
HbA1C (%)	−0.00	>0.05	0.36	0.01	−0.01	>0.05
Cortisol (µg/dL)	0.36	0.02	−0.13	>0.05	−0.08	>0.05
Insulin (µlu/mL)	0.21	>0.05	0.19	>0.05	−0.01	>0.05

MetS: group with metabolic syndrome; Pre-MetS: group with risk of metabolic syndrome; WC: waist circumference; HC: hip circumference; SBP: systolic blood pressure; DBP: diastolic blood pressure; MCV: mean corpuscular volume; MCH: mean corpuscular hemoglobin; MCHC: mean corpuscular hemoglobin concentration; FPG: fasting plasma glucose; TG: triglyceride; HDL: high-density lipoprotein; LDL: low-density lipoprotein; HbA1C: glycated hemoglobin; r_p_: Pearson’s r correlation; *p*-value: significance level.

**Table 4:** Women’s menstruation status and metabolic syndrome status.

Menstruation	MetS (n = 47)		Pre-MetS (n = 64)		Control (n = 122)		All (n = 233)	
	n	%	n	%	n	%	n	%
Yes	13	27.7	22	34.4	47	38.5	82	35.2
No	34	72.3	42	65.6	75	61.5	151	64.8

MetS: group with metabolic syndrome; Pre-MetS: group with risk of metabolic syndrome; n: number of women; %: percent of women.

**Table 5:** The women’s menstruation status and Pb-B levels (µg/dL) with regard to metabolic syndrome status.

Menstruation	MetS (n = 47)		Pre-MetS (n = 64)		Control (n = 122)		F_(2,224)_	*p*-Value *
	M	SD	M	SD	M	SD		
No	5.37	1.83	7.46	2.88	8.14	3.74	0.632	0.532
Yes	5.63	1.87	6.11	2.16	7.80	5.31		

MetS: group with metabolic syndrome; Pre-MetS: group with risk of metabolic syndrome; M: mean; SD: standard deviation; F: statistical value; *p*-value: significance level; * two-way ANOVA.

**Table 6:** Pb-B levels (µg/dL) with regard to stimulants (alcohol, caffeine, tobacco).

Stimulants	MetS (n = 47)		Pre-MetS (n = 64)		Control (n = 122)		F_(2, 176)_	*p*-Value *
	M	SD	M	SD	M	SD		
Alcohol								
No	5.04	1.81	6.80	2.69	6.94	2.61	0.816	0.439
Yes	6.32	1.66	7.47	2.80	9.22	6.40		
Caffeine								
No	0.00	0.00	0.00	0.00	0.00	0.00	− **	− **
Yes	5.44	1.82	6.99	2.71	8.01	4.38		
Tobacco								
No	5.40	1.85	7.00	2.64	8.10	4.91	0.971	0.381
Yes	6.11	1.84	7.30	3.09	6.39	1.82		

MetS: group with metabolic syndrome; Pre-MetS: group with risk of metabolic syndrome; M: mean; SD: standard deviation; F: statistical value; *p*-value: significance level; * two-way ANOVA; ** lack of answer ‘No’results in no comparative analysis of the impact of caffeine consumption.

**Table 7:** Metabolic syndrome status and the severity of climacteric and depressive symptoms.

Scale/Subscale	MetS (n = 47)	Pre-MetS (n = 64)	Control (n = 122)	F	*p*-Value	η^2^
	M (SD)	M (SD)	M (SD)			
KI	14.49 (9.22)	14.38 (9.59)	13.55 (8.86)	0.28	0.76	0.002
BDI	6.04 (5.51)	9.70 (8.23)	8.08 (6.85)	3.69	0.03	0.031

MetS: group with metabolic syndrome; Pre-MetS: group with risk of metabolic syndrome; KI: Blatt–Kupperman Index; BDI: Beck Depression Inventory; M: mean; SD: standard deviation; F: statistical value; *p*-value: significance level.

**Table 8:** Correlation between Pb-B levels (µg/dL) and depressive symptoms with regard to metabolic syndrome status.

Depressive Symptoms	MetS (n = 47)		Pre-MetS (n = 64)		Control (n = 122)	
	r_p_	*p*-Value	r_p_	*p*-Value	r_p_	*p*-Value
BDI	−0.064	0.676	−0.084	0.508	0.003	0.971

MetS: group with metabolic syndrome; Pre-MetS: group with risk of metabolic syndrome; BDI: Beck Depression Inventory; M: mean; SD: standard deviation; r_p_: Pearson’s r correlation; *p*-value: significance level.
